# Survey of remote consultations in psychiatry during the SARS-CoV-2 outbreak

**DOI:** 10.1192/bjo.2021.704

**Published:** 2021-06-18

**Authors:** Nusra Khodabux, Satheesh Gangadharan, Samuel Tromans, Avinash Hiremath

**Affiliations:** Leicestershire partnership NHS Trust

## Abstract

**Aims:**

To compare the usage of remote consultations before and after the first wave of the SARS-CoV-2 outbreak and explore mental health workers’ views on the usage of telemedicine.

**Method:**

An online questionnaire survey was developed, and disseminated to mental healthcare professionals via e-mail and social media. Quantitative data were analysed using descriptive statistics and qualitative data were analysed using Braun and Clarke's six step procedure for thematic analysis.1

**Result:**

There were 40 responses from mental healthcare professionals of varying grades from different sub-specialties, predominantly from the UK. Compared to before the SARS-CoV-2 outbreak, there was an increase in usage of telephone (9(22.5% to (29)72.5%) and video consultations (4(10%) to 17(42.5%)). Respondents reported an increase in virtual MDTs (35(87.5%) during the pandemic, 9(22.5%) pre-pandemic).

Based on a 5-point Likert scale, the mean technical quality of telephone consultations was 3.56/5 (Range 2-5), with 75% rating telephone consultations as not being as good as face-to-face consultations. The mean technical quality of video consultations was 3.58/5 (Range 2-5), with 63% rating video consultations as not being as good as face-to-face consultations. 25 (62.5%) respondents felt comfortable using telephone consultations during the pandemic, 20(50%) felt comfortable using video consultations. Recurring themes identified from the qualitative data regarding reasons for the technical quality ratings were: connection issues, poor infrastructure and security concerns.

Nine (23%) respondents felt that using video conferencing consultations had a detrimental impact on the mental health of patients while 14(35%) felt that telephone consultations had a detrimental impact on patients’ mental health. Recurring themes for health practitioners’ perceived effect of the use of telemedicine on patients’ mental heath were the loss of personal touch and reduced patient engagement.

**Conclusion:**

There was a substantial increase in usage of remote consultations during the first wave of the SARS-CoV-2 pandemic among mental healthcare professionals. The results reported in the present study suggest there are numerous barriers to the use of telemedicine in psychiatry, which require future exploration, ideally through interview or ethnographic studies.
